# The *Mycobacterium tuberculosis* Rv0132c Gene Product Mtb‐FGD2 Can Act as an F_420_ ‐Dependent Glucose Dehydrogenase

**DOI:** 10.1002/prot.70139

**Published:** 2026-04-21

**Authors:** Adewale V. Aderemi, Matthew Snee, Richard B. Tunnicliffe, Linus O. Johanissen, Matthew J. Cliff, Colin W. Levy, Derren J. Heyes, Marina Golovanova, Thomas A. Jowitt, Sam Hay, Andrew W. Munro, Jonathan P. Waltho, David Leys

**Affiliations:** ^1^ Manchester Institute of Biotechnology, Department of Chemistry University of Manchester Manchester UK; ^2^ Osun State University Osogbo Osun State Nigeria; ^3^ School of Biological Sciences, Faculty of Biology, Medicine and Health, Michael Smith Building University of Manchester Manchester UK; ^4^ Biomolecular Analysis Core Facility, Faculty of Biology, Medicine and Health, Michael Smith Building University of Manchester Manchester UK; ^5^ School of Biosciences The University of Sheffield Sheffield UK

**Keywords:** F_420_, glucose dehydrogenase, *Mycobacterium tuberculosis*, redox enzyme, Rv0132c

## Abstract

The role of the cell envelope‐associated Rv0132c/FGD2 from *Mycobacterium tuberculosis* has long been a subject of debate. Importantly, FGD2 is found only in pathogenic mycobacteria, making it a potential drug target. While some suggest it functions as a glucose‐6‐phosphate dehydrogenase, others propose it acts instead as an F_420_‐dependent hydroxy‐mycolic acid dehydrogenase—an activity linked to cell‐wall remodeling and inhibition by the anti‐tubercular drug pretomanid. Yet, direct evidence for either activity has been lacking. Here, we heterologously express and purify active Mtb‐FGD2, and demonstrate that the enzyme binds the F_420_ cofactor with nanomolar affinity. Crystal structures for both the *apo‐*form and the F_420_ complex reveal that the Mtb‐FGD2 active site architecture is consistent with sugar substrates but notably lacks a phosphate‐binding pocket. Biochemical assays confirm that Mtb‐FGD2 functions efficiently as an F_420_‐dependent glucose dehydrogenase in vitro. Computational docking combined with molecular dynamics simulations further supports the formation of a catalytically plausible β‐D‐glucose:F_420_ ternary complex. When coupled to other F_420_‐dependent enzymes, Mtb‐FGD2 readily supports glucose‐driven F_420_.H_2_‐dependent oxidoreductase activity. Our data thus suggest that the Mtb‐FGD2 provides reduced F_420_.H_2_ in a glucose‐dependent manner to support mycobacterial F_420_.H_2_‐dependent oxidoreductases in the cell envelope.

**Abbreviations**
AOXPPP: alternative oxidative phase of the pentose phosphate pathway
ESAT‐6: early‐secreted antigenic factor‐6
FGD: F_420_‐dependent glucose‐6 phosphate dehydrogenase
fHMAD: F_420_‐dependent hydroxy‐mycolic acid dehydrogenase
HEPES: 4‐(2‐hydroxyethyl)‐1‐piperazineethanesulfonic acid
ITC: isothermal titration calorimetry
KPi: potassium phosphate
MS: mass spectrometry
Mtb:
*Mycobacterium tuberculosis*

MTBC:
*Mycobacterium tuberculosis* complex
NADP: nicotinamide adenine dinucleotide phosphate
Nano‐ESI: nano‐electrospray ionization
PDIM: phthiocerol dimycocerosates
PKR: phthiodiolone ketoreductase
SAD: single‐wavelength anomalous diffraction
SDS‐PAGE: sodium dodecyl‐sulfate polyacrylamide gel electrophoresis
SLS: static light scattering
TB: tuberculosis
TCEP: tris(2‐carboxyethyl)phosphine
[TeW_6_O_24_]^6−^
: tellurium‐centered Anderson–Evans poly‐oxotungstate
TFS: tryptophan fluorescence spectroscopy
Tfu‐FNO: thermostable F_420_:NADPH oxidoreductase from *T. fusca*

*T*_m_
: melting temperature

## 1 Introduction

Tuberculosis (TB) continues to be a life‐threatening disease of man, accounting for a large proportion of mortalities arising from a single infectious agent worldwide [1]. The majority of these deaths are a result of the emergence of resistance to the traditional (frontline) anti‐TB regimens [2]. For instance, in 2021 alone, about 190 000 deaths resulting from multi‐drug resistant cases were reported [3]. This problem is particularly pronounced in the developing economies of the world, where treatment modalities such as directly observed short courses are not yielding the expected outcomes [4]. Even for relatively “novel” anti‐TB drugs, such as aminosalicylic acid and linezolid, the problem of adverse drug reactions implies that better‐tolerated new anti‐TB agents will be needed [5]. Clearly, next‐generation drug candidates would likely need to utilize mechanisms of action that are altogether different from those employed by the drugs currently in use for TB treatment. To this end, enzymes that utilize the unusual deazaflavin cofactor (F_420_) could present promising alternatives, particularly because humans, the only known obligate host of Mtb [6], lack this coenzyme [7]. At least twenty‐eight F_420_‐utilizing enzymes have been predicted [8] to be present in Mtb but, apart from Ddn [9], FGD1 [10] and Rv1155 [7], these have not been fully characterized.

**FIGURE 1:**
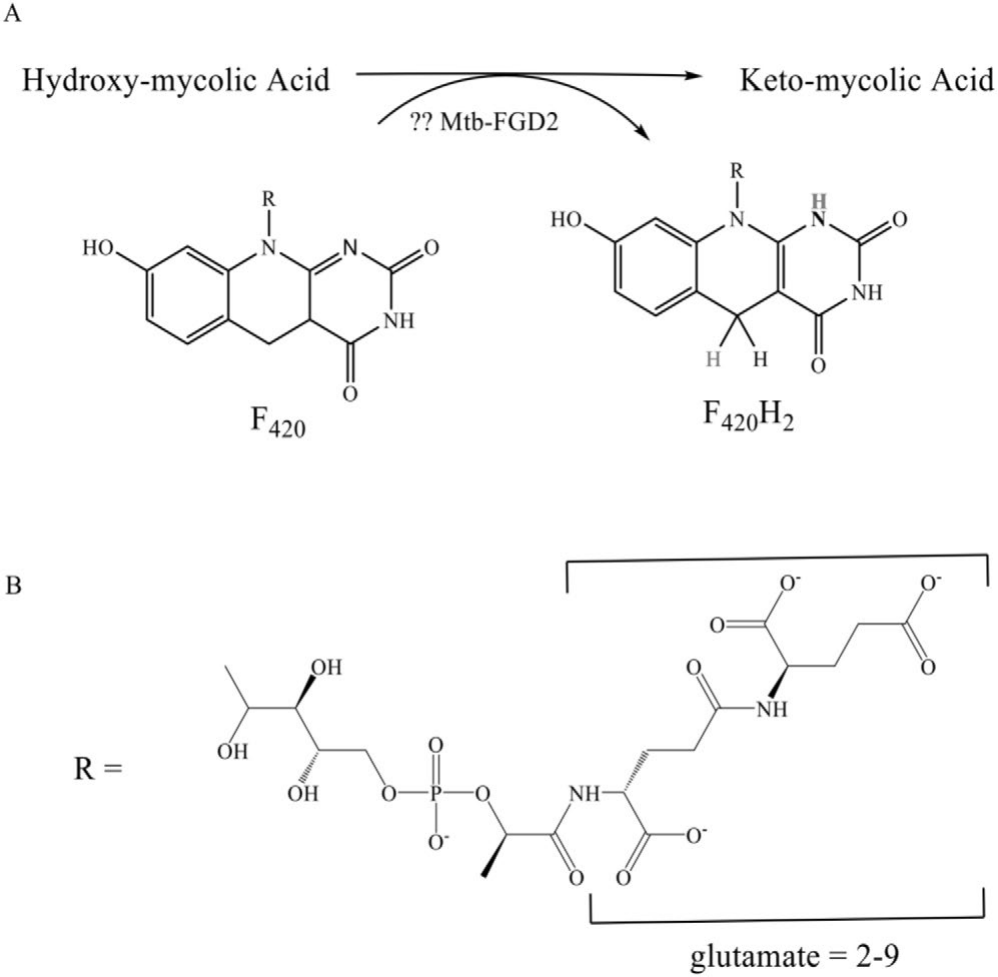
The proposed functional role of Mtb‐FGD2 (Rv0132c). (A) Rv0132c/Mtb‐FGD2 is implicated in the conversion of hydroxy‐mycolic acid to keto‐mycolic acid in the Mtb cell wall, with the corresponding reduction of the co‐factor F_420_ to F_420_.H_2_ [12]. (B) The R‐group in F_420_ consists of a ribityl‐phospho‐lactyl‐glutamate tail, containing 2–9 glutamate residues.

The Rv0132c‐encoded Mtb‐FGD2 is an example of an F_420_‐utilizing enzyme in Mtb whose biological role has been a subject of controversy. It was originally proposed to be a second F_420_‐dependent G6P dehydrogenase, but was later shown to lack this functionality [11]. Shortly after, Mtb‐FGD2 was implicated to be involved in the conversion of hydroxy‐mycolic acid to keto‐mycolic acid in the Mtb cell wall (Figure 1) [12]. Mtb‐FGD2 was thus re‐classified as an F_420_‐dependent hydroxy‐mycolic acid dehydrogenase (fHMAD). While the enzyme appears to have a role to play in the cell wall (as it possesses a unique N‐terminal TAT signaling sequence [11]), no direct evidence to support hydroxy‐mycolic acid dehydrogenase activity has been reported. Instead, the reclassification to an fHMAD is based on a causal relationship between the introduction of the newly approved anti‐tubercular agent, pretomanid, and reduction in the expression level of the heterologously expressed Rv0132c gene in *Mycobacterium smegmatis* . Since its re‐annotation as an fHMAD enzyme [12], little has been done to further characterize Mtb‐FGD2 as a drug target. To gain insight into the Mtb‐FGD2 catalytic and ligand binding properties, as well as support future drug development studies, we show that the enzyme can be successfully produced in *E. coli* , using GroEL/ES as chaperones, similar to Mtb‐FGD1 [13]. We report the crystal structures of Mtb‐FGD2 in both *apo‐*form and in complex with the F_420_ cofactor, using tellurium‐centred Anderson‐Evans poly‐oxotungstate [TeW_6_O_24_]^6−^ as a crystallization additive [14, 15, 16]. Combined with docking and molecular dynamics studies, these structures rationalize our observation that Mtb‐FGD2 can act as a F_420_‐dependent D‐glucose dehydrogenase.

## 2 Results

### 2.1 Expression, Purification and Cofactor Binding of Mtb‐FGD2

**FIGURE 2:**
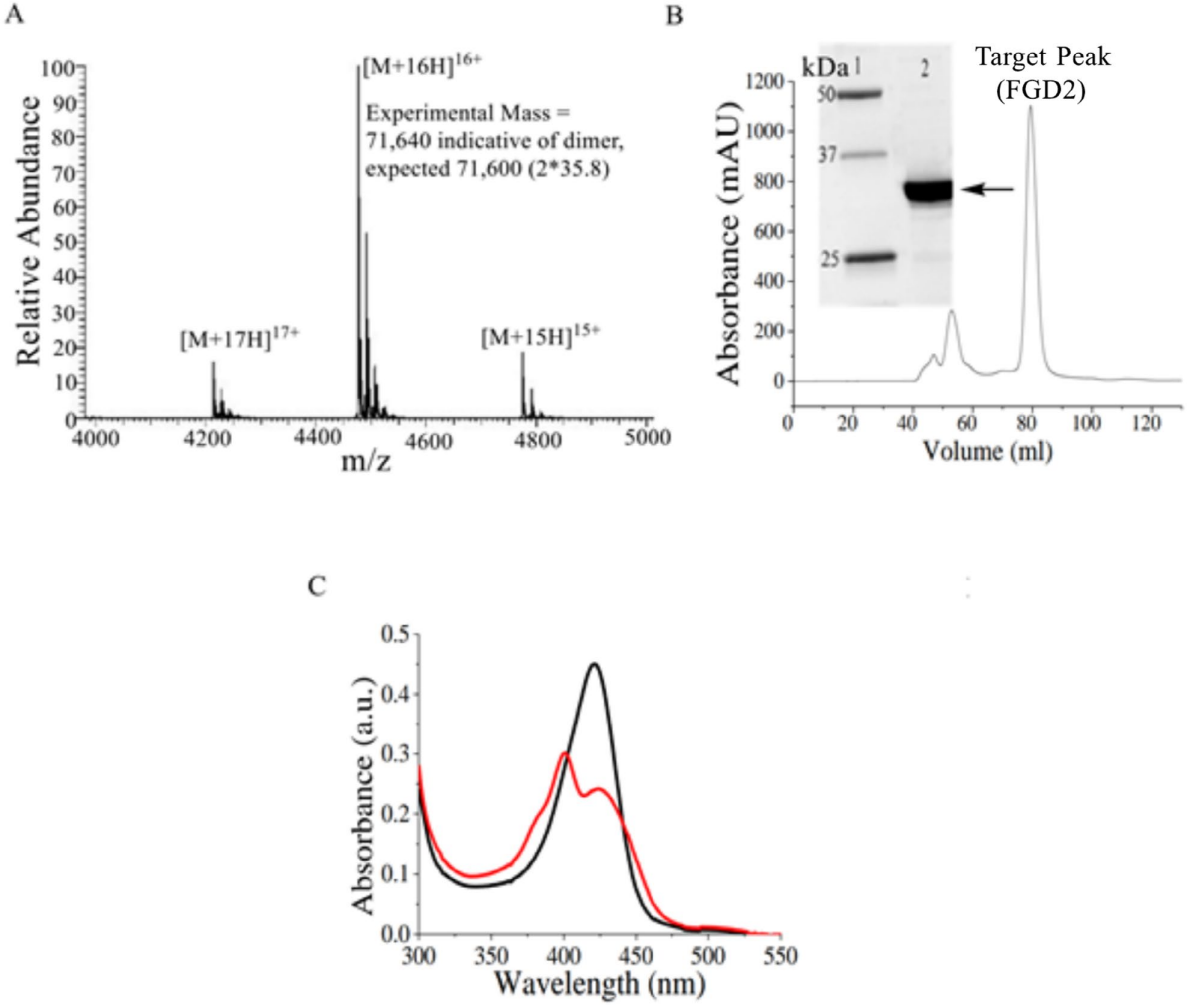
Mtb‐FGD2 purification and spectral analysis. (A) Nano‐ESI MS with estimated mass corresponding to a Mtb‐FGD2 dimer (m/z = 71 640 Da) compared to the predicted 71 600 Da. (B) Gel filtration profile of Mtb‐FGD2 with the SDS‐PAGE analysis (inset) showing a sample of the purified enzyme in lane 2. (C) UV–Vis absorption spectra of free F_420_ (*black*) vs. Mtb‐FGD2‐bound F_420_ (*red*).

**FIGURE 3:**
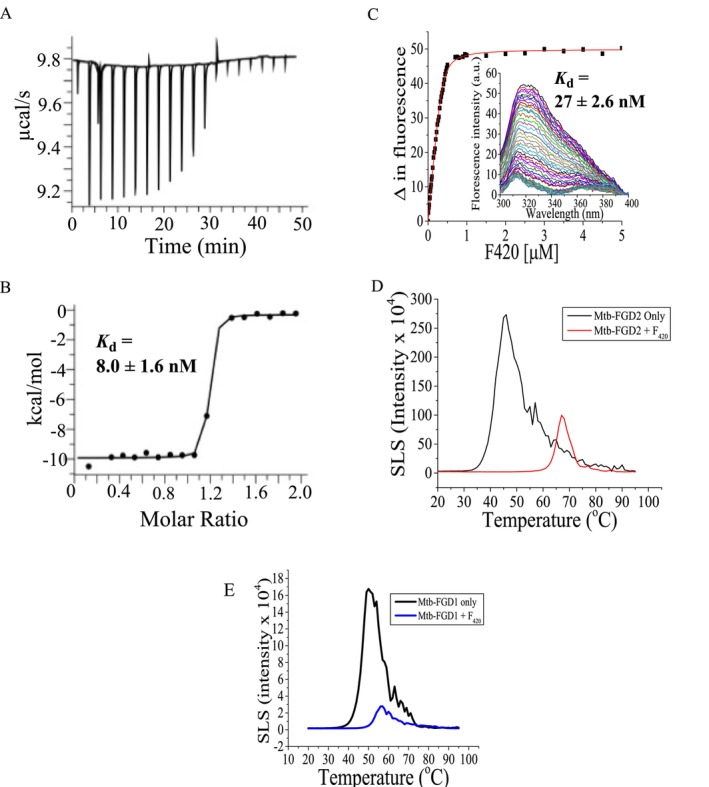
F_420_ binding to Mtb‐FGD2. (A) ITC measurement of F_420_ binding to Mtb‐FGD2 showing the time course of heat evolution for the titration of Mtb‐FGD2 (250 μM) against F_420_ (25 μM). (B) ITC thermogram showing the peak integration as a function of the molar ratio of the ligand to protein. The derived *K*_*d*_ value was 8.0 ± 1.6 nM from 2 replicates. (C) TFS measurement of F_420_ binding to Mtb‐FGD2 showing the change in fluorescence versus F_420_ concentration after normalization against intrinsic fluorescence. Approximately 1 μM of Mtb‐FGD2 was titrated against an increasing concentration of F_420_ (0–5 μM). The derived *K*_*d*_ value was 27.0 ± 2.6 nM from 2 replicates. Inserts are the corresponding emission spectra (300–400 nm), obtained at an excitation wavelength of 285 nm. (D) Static Light Scattering (SLS) measurement of *T*_m_ for F_420_ (70 μM) binding to Mtb‐FGD2 (14 μM) using the protein stability screening platform Uncle. (E) The equivalent data to panel D for Mtb‐FGD1 (14 μM).

The mature form of Mtb‐FGD2, lacking the Tat signal consisting of amino acids 1–38, was successfully expressed using *E. coli* . This was achieved with the use of a GroEL/ES‐expressing *E. coli* BL21 (DE3) strain. Mtb‐FGD2 was found to express to a high level, with ~75 mg of purified enzyme obtained per liter of culture. Following purification, the enzyme was confirmed to be a dimer in solution confirmed using Nano‐Electrospray Ionization (Nano‐ESI) Mass Spectrometry (Figure 2A,B). When F_420_ is added to the purified *apo‐*enzyme, two peaks were observed in the corresponding UV–Vis spectrum, at 400 nm and 440 nm, as opposed to the 420 nm absorbance maximum of free F_420_ (Figure 3C). The F_420_ spectral perturbation following protein binding has been previously noted in other F_420_—dependent enzymes, including Mtb‐FGD1 [11]. The binding affinity of Mtb‐FGD2 for F_420_ was determined using both isothermal titration calorimetry (ITC) and intrinsic tryptophan florescence spectroscopy (TFS) (Figure 3A–C). Both reveal the enzyme binds to F_420_ with *K*_d_ values in the nano‐molar range (8.0 ± 1.6 nM with ITC and 27.0 ± 2.6 nM with TFS). The melting temperature (*T*_m_) of Mtb‐FGD2 was determined both for the *apo*‐ and holoenzyme forms (Figure 3D). In the *apo*‐form, the *T*_m_ was approximately 37°C, but F_420_ addition led to a dramatic increase in Mtb‐FGD2 thermostability (*T*_m_ of 65°C), giving a Δ*T*_m_ of 28°C, compared to Mtb‐FGD1 with a ΔT_m_ of 20°C (Figure 3E, from 47°C to 67°C) upon addition of F_420_.

### 2.2 Crystal Structures of Mtb‐FGD2

**FIGURE 4:**
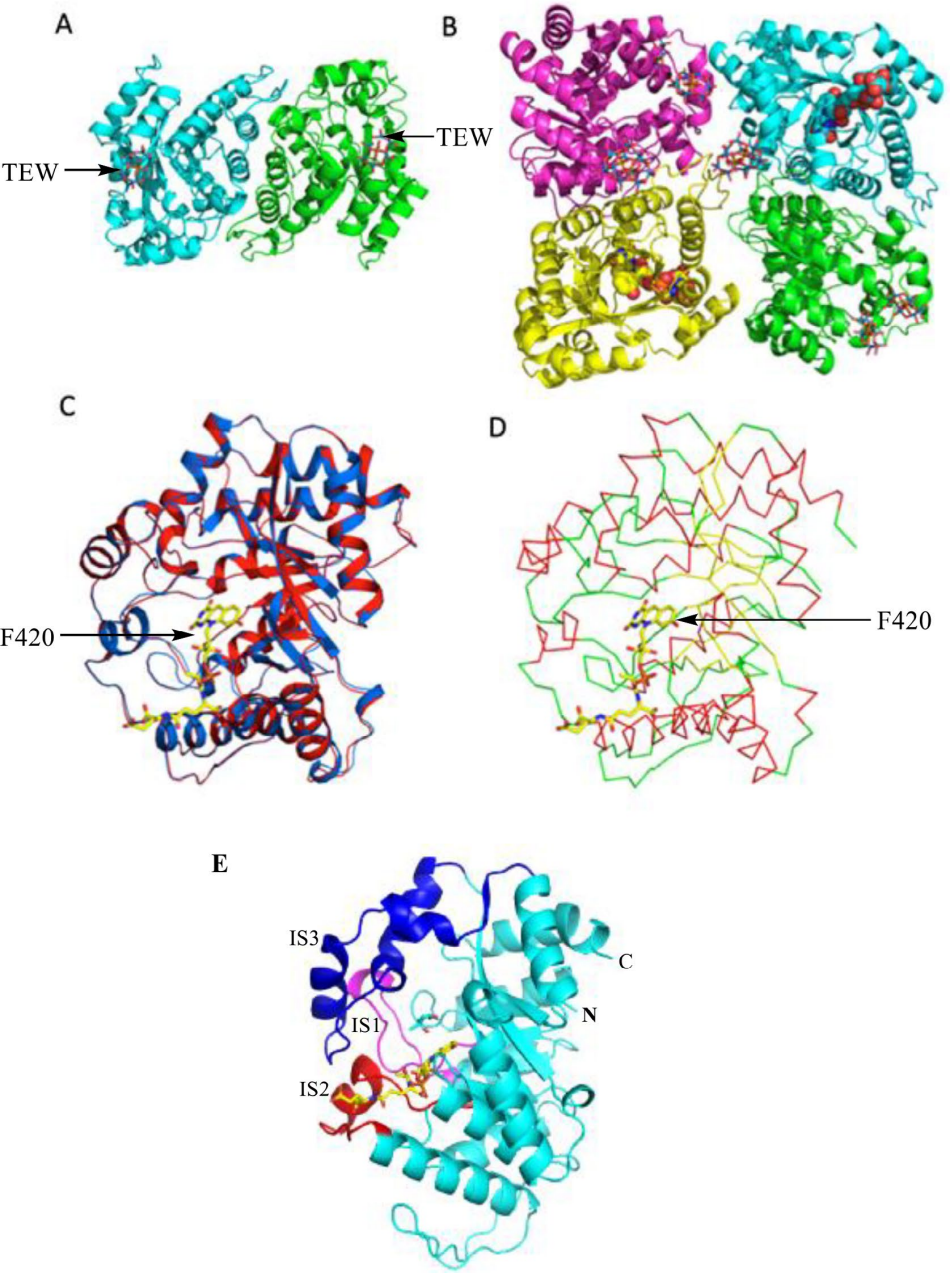
Structures of F_420_‐free and F_420_‐bound Mtb‐FGD2. (A) Cartoon diagram of *apo*‐Mtb‐FGD2 dimer, bound to TEW (shown in sticks). (B) Cartoon view of F_420_‐bound Mtb‐FGD2, showing the 4 monomers and the relative positioning of TEW (in sticks) at the inter‐dimeric interface and on the surfaces of chain (A and C), and the two bound F_420_ (shown in spheres) in chain B and D. (C) Cartoon view of the structural alignment of chain B of *apo*‐FGD2 (*blue*) with chain B of F_420_‐bound (*red*) monomer (r.m.s.d = 0.189 Å). (D) Ribbon view of Mtb‐FGD2 monomer (Chain B) showing the secondary elements (α‐helix in *red*, β‐sheet in *yellow* and the loops in *green*). (E) Ribbon view of the protein showing the 3 flexible insertion segments IS1 (magenta), IS2 (red) and IS3 (blue).

Initial efforts at crystallizing Mtb‐FGD2 yielded poorly diffracting crystals. However, the introduction of TEW (Tellurium‐centered Anderson−Evans Polyoxotungstate [TeW_6_O_24_]^6−^) as a crystallization additive [16] yielded well‐diffracting *apo*‐crystals after 1–2 days of incubation at 4°C. Crystals of the F_420_‐bound form appeared more slowly over 3–4 weeks. Both the *apo*‐ and F_420_‐bound Mtb‐FGD2 crystals belonged to space group P2_1_2_1_2_1_ and yielded diffraction data to 1.45 Å and 2.35 Å respectively. The structure of *apo*‐Mtb‐FGD2 (PDB accession number **9FP4**) (Figure 4A) was solved taking advantage of the TEW anomalous signal. Following model building and refinement, the corresponding model was used to solve the F_420_ complex structure (PDB accession number **9FFP**). The data collection and refinement statistics are shown in Table 5. A single dimer is present in the asymmetric unit of the *apo* structure, while two dimers (i.e., the A/B and C/D monomer pairs) are present in the F_420_‐bound asymmetric unit (Figure 4B). In both *apo* and F_420_‐bound structures, electron density corresponding to bound TEW was readily observed. In the F_420_‐bound structure, electron density for F_420_ is clearly visible for one monomer from each dimer (i.e., chains B and D) (Figure 4B), along with some evidence for the cofactor in the other chains at much lower occupancy. The Mtb‐FGD2 monomer comprises an (αβ)_8_ TIM‐barrel, showing the unusual non‐prolyl cis‐peptide bond located near the *re*‐face of the F_420_‐isoalloxazine ring [10, 17, 18, 19] where the active site is located [17].

### 2.3 F_420_ Binding in the Active Site of Mtb‐FGD2

In Mtb‐FGD2, the non‐prolyl cis‐peptide bond is located between Gly111 and Val112 in the β3 strand of the enzyme (Figure 4D), where it serves to support binding of the 3‐membered F_420_‐ring system. Similar behavior is found in other members of the luciferase family with an F_420_‐binding active site, including Mtb‐FGD1 (Ser73—Val74), [10] Mer (Gly61—Val62), [17] and Adf (Cys72—Ile73) [20]. The (*αβ*)_8_ TIM (triosephosphate isomerase) barrel structure of the F_420_‐bound protein is such that the cofactor lies within the core formed by the β‐sheets, surrounded externally by the α‐helices (Figure 4D). The TIM barrel is a type of protein fold that is made of eight alternating alpha‐helices (*α*) and beta‐strands (*β*), forming a barrel shape. The active site is often located at the C‐terminal end of the beta‐strands. As with other luciferase enzymes, the active site is capped by thre highly flexible, insertion segments, namely IS1 (Ala75—Pro92), IS2 (Leu143—Tyr161), and IS3 (Asp270—Asp317) [10, 18], with the ring system of the F_420_ cofactor buried in the active site and the polyglutamate tail extending outwards towards the solvent (Figure 4C,D). Only two glutamate residues are clearly visible in the electron density of the bound F_420_ in chains B and D. An alignment of the *apo*‐Mtb‐FGD2 (chain B), with that in complex with F_420_ (chain B), showed a significant similarity between the two monomers (r.m.s.d of 0.189 Å), but with a noticeable inward shift in α8‐helix and the insertion points IS2 and IS3 in chain B of the Mtb‐FGD2:F_420_ complex (Figure 4C).

### 2.4 F_420_ Binding in Mtb‐FGD2 Versus Mtb‐FGD1

**FIGURE 5:**
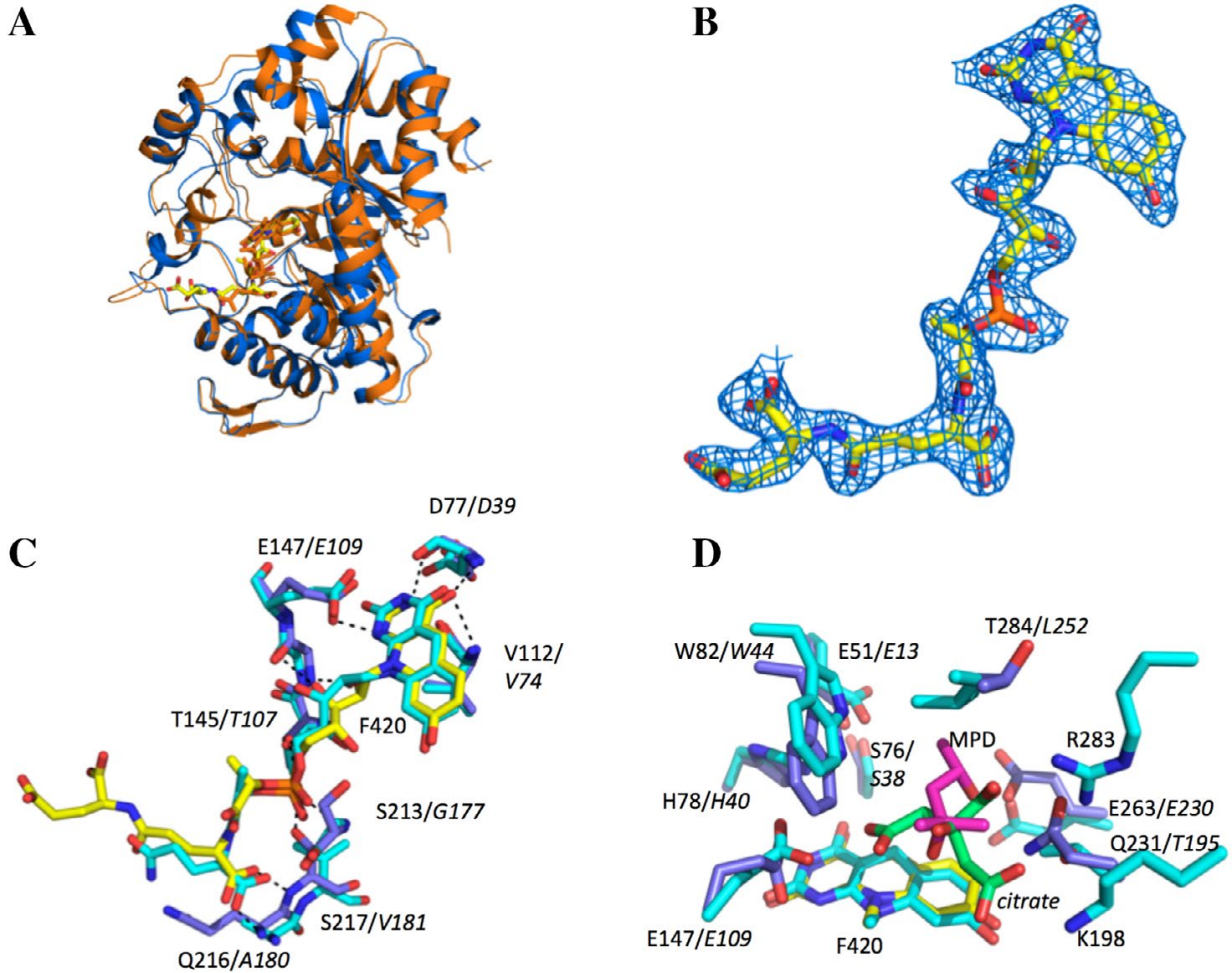
Mtb‐FGD2 ligand binding compared with Mtb‐FGD1. (A) Cartoon view of structural alignment of Mtb‐FGD2‐F_420_ (in *orange, with F*_*420*_ *in yellow*) with Mtb‐FGD1‐F_420_ (colored *in blue*). (B) 2f_o_f_c_ electron density contoured at 1 sigma corresponding to the bound F420 cofactor in chain A. (C) Comparison of key protein ligand interactions made between F420 and respectively Mtb‐FGD2 (protein in blue, F420 in yellow) and Mtb‐FGD1 (all in cyan). Hydrogen bonding interactions shown in dotted lines. Amino acids labels for Mtb‐FGD1 *in italics*. (D) Comparison of key protein ligand interactions made between crystallization conditions derived molecules and respectively Mtb‐FGD2 chain B (protein in blue, MPD in magenta) and Mtb‐FGD1 (protein in cyan, citrate in green). Amino acids labels for Mtb‐FGD1 *in italics*.

A pairwise alignment between monomers of the individual monomers A, B, C, and D in the asymmetrical unit of the Mtb‐FGD2:F_420_ complex reveals r.m.s.d. values ranging from 0.036 Å to 0.111 Å. The equivalent comparison of the Mtb‐FGD2‐F_420_ complex (chain B) with the Mtb‐FGD1:F_420_ complex (Chain B, PDB Accession Number 3B4Y) [10] showed significant similarities in terms of both the overall fold (excluding the polyhistidine tag on the Mtb‐FGD1:F_420_ complex, r.m.s.d of ~1.0 Å; Figure 5A), and the F_420_ cofactor interactions formed. For instance, the butterfly‐like conformation of the cofactor, reported for Mtb‐FGD1:F_420_ complex, arising from the steric effects of its interaction with active site residues [10], is also observed in Mtb‐FGD2:F_420_ complex (Figure 5C). Additionally, the key hydrogen bond interactions between the enzyme and the F_420_ ring system (involving Mtb‐FGD2 Asp77, Val112, Glu147, Asn150) are similar to those in the Mtb‐FGD1:F_420_ complex (involving corresponding Mtb‐FGD1 Asp39, Val74, Glu109, Asn112). The ribityl portion of the cofactor, which forms hydrogen bonds with main chain of Gly144 and Gly146 in the Mtb‐FGD2:F_420_ complex, is also within hydrogen bond distance with corresponding Gly106 and Gly108 in the Mtb‐FGD1:F_420_ complex (Figure 5C). Few additional interactions are observed for the Mtb‐FGD2:F_420_ complex, including a hydrogen bond between Gln231 and the F_420_ ring system. Therefore, comparison of the F_420_‐complex structures does not rationalize why F_420_ binds much more tightly to Mtb‐FGD2 (8–27 nM) than to Mtb‐FGD1 (4.5 μM) [10].

### 2.5 Substrate Binding Site Architecture of Mtb‐FGD2

An examination of the Mtb‐FGD2 active site residues of the F_420_‐bound monomers B and D shows near identical conformation and reveals significant similarities with the Mtb‐FGD1 active site (Figure 5D). Residues that interact with the C1 hydroxyl group of G6P in Mtb‐FGD1, namely Glu13, His40, and Trp44, correspond to Mtb‐FGD2 Glu51, His78, and Trp82, and these adopt a similar configuration. However, Mtb‐FGD2 lacks suitable equivalents to the Mtb‐FGD1 charged residues Lys198, Lys259, and Arg283 involved in the binding of the phosphate moiety of G6P. In the Mtb‐FGD2 structure, similar positions are occupied by side chains of Asp234, Pro291, and Asn311, respectively. Additionally, three water molecules are located within hydrogen bonding distance of Glu263, a residue previously proposed to play a role in HMA binding [19]. Finally, there is electron density resembling 2‐methyl‐1,3‐propanediol (MPD), derived from the crystallization condition used [21], occupying the active site cavity and sandwiched between Trp82 and the F_420_ cofactor (Figure 5D). This cavity is similarly occupied by citrate in Mtb‐FGD1, which is postulated to mimic the G6P binding pose [10].

### 2.6 Mtb‐FGD2 Substrate Profiling

**FIGURE 6:**
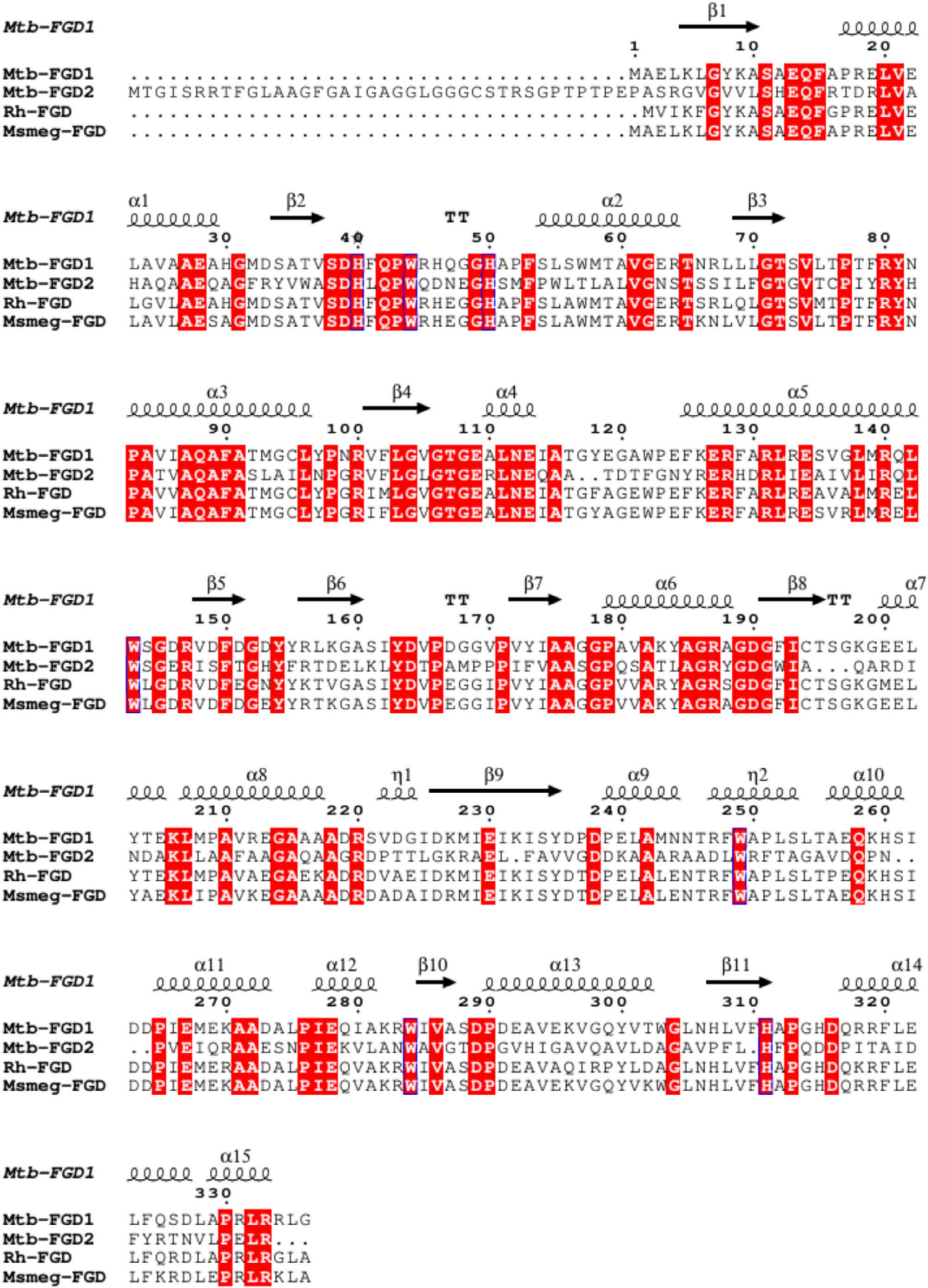
Multiple sequence alignment of Mtb‐FGD2 homologues across relevant species. Numbering and secondary structural elements are with respect to Mtb‐FGD1. Residues involved with the G6P substrate binding (E13, H40, W44, K198, K259 and R283) [10] are as shown in black asterisks, with only Mtb‐FGD2 lacking the residues for binding the phosphate moiety of G6P. Figure was drawn with the use of ESpript version 3.0 [23].

To gain insight into the likely substrate for Mtb‐FGD2, the sequence was compared with closest relative Mtb‐FGD1 [10] and two other G6P–utilizing enzymes from *M. smegmatis* (Ms) [22] and *Rhodococcus jostii* (Rh) [18] (Figure 6). This alignment confirms the strong conservation of residues involved in the binding of the glucose moiety of G6P, but charged residues involved in binding the phosphate moiety of G6P are present only in Mtb‐FGD1, Rh‐FGD, and Ms‐FGD. Together with our structural insights, this observation points to the possibility of a non‐phosphorylated sugar as substrate for Mtb‐FGD2. The enzyme was therefore screened for catalyzing F_420_ reduction with a panel consisting of simple sugars, sugar derivatives and a range of secondary alcohols (Table 1). The screen was carried out by following the substrate‐dependent decrease in F_420_ absorbance at 420 nm as an indicator of Mtb‐FGD2 mediated redox activity. This substrate profiling showed that Mtb‐FGD2 displayed significant activity with a limited set of sugars. Highest activity was observed with D‐glucose, with the related simple sugars D‐ribose, D‐arabinose, D‐galactose, L‐arabinose and D‐mannose (in order of decreasing activity levels) supporting only modest F_420_ reduction in comparison (Table 1). No activity was observed with the other sugars tested, or with any of the secondary alcohols. When added to D‐glucose‐dependent F_420_ reduction assays, none of the inactive compounds displayed any inhibition, suggesting these do not bind to the enzyme at the concentrations used.

**TABLE 1:** Substrate profiling for Mtb‐FGD2.

S/No	Compound screened	*K*_m_ (μM)	*k*_cat_ (s^−1^)	*k*_cat_/*K*_m_ (M^−1^ s^−1^)
1	D‐Glucose	220 ± 20	0.40 ± 0.07	1800
2	D‐Ribose	190 ± 8	0.15 ± 0.01	800
3	D‐Galactose	150 ± 20	0.050 ± 0.004	340
4	L‐Arabinose	140 ± 30	0.04 ± 0.01	280
5	D‐Mannose	580 ± 120	0.014 ± 0.01	25
6	D‐Arabinose	642 ± 13	0.04 ± 0.03	60
7	D‐Xylose	No activity	No activity	No activity
8	D‐Lyxose	No activity	No activity	No activity
9	D‐Glucosamine 6‐phosphate	No activity	No activity	No activity
10	Glyceraldehyde 3‐phosphate	No activity	No activity	No activity
11	Glucose 6‐phosphate	No activity	No activity	No activity
12	Ribose 1‐phosphate	No activity	No activity	No activity
13	Ribose 5‐phosphate	No activity	No activity	No activity
14	D‐lactate	No activity	No activity	No activity
15	Trehalose	No activity	No activity	No activity
16	Isopropanolol	No activity	No activity	No activity
17	*N*‐acetyl‐D‐glucosamine	No activity	No activity	No activity
18	Sorbitol	No activity	No activity	No activity
19	Xylitol	No activity	No activity	No activity
20	Glycerol	No activity	No activity	No activity

*Note:* The table shows the results of the steady‐state kinetics carried out on a range of simple and derived sugars, secondary alcohols, and other compounds to test their activities against Mtb‐FGD2.

### 2.7 Docking of D‐Glucose and Molecular Dynamics Simulations

**FIGURE 7:**
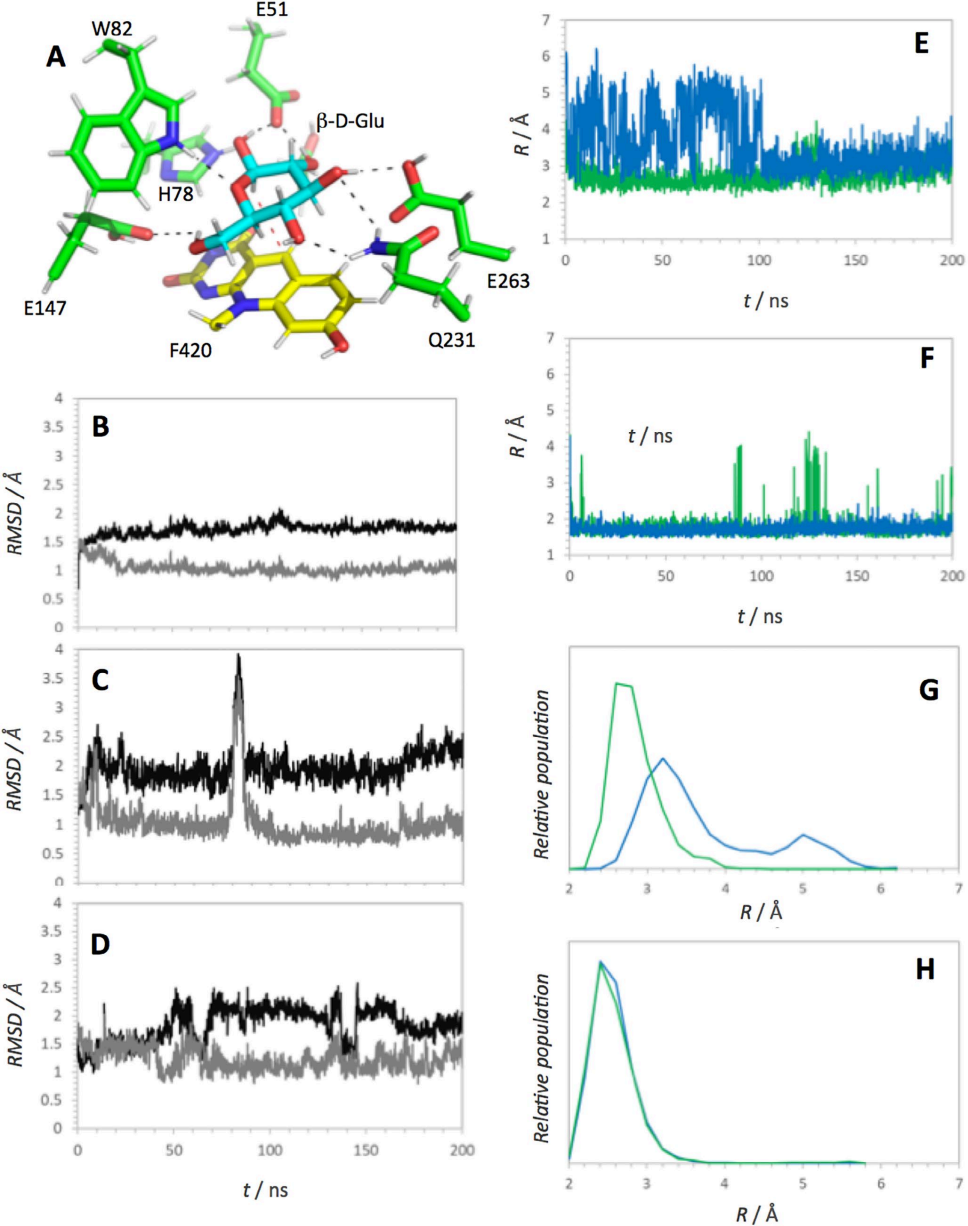
Modeling of the β‐D‐glucose Mtb‐FGD2 complex. (A) Stick model representation of β‐D‐glucose bound to Mtb‐FGD2 –active site observed during the MD simulations. The principal active site residues Glu51, His78 and Trp82 are in close proximity to the substrate C1 position. The β‐D‐glucose C2 and C4 hydroxyl groups make hydrogen bond contacts with Gln231 and Glu263. (B–D) Root mean squared deviations (RMSDs) from the starting structure (black lines) and the average structure (gray lines) for 200 ns MD simulation of FGD2 reactive complex: (B) RMSD of protein heavy atoms (i.e., not including hydrogens), (C) RMSD of chain A active site residues and (D) RMSD of chain B active site residues; active site residues are simply defined as those with at least one atom within 4 Å of the substrate in the starting structure. The RMSD from the average was computed after structural alignment to the heavy atoms of the starting structure. (E–H) Distances for hydride and proton transfers, for chain A (blue) and chain B (green): Distance vs time plots for (E) C—H and (F) O—H distances and relative populations for (G) C—H and (H) O—H distances.

Following substrate profiling, attempts at co‐crystallization or soaking of the *apo‐* and F_420_‐bound Mtb‐FGD2 crystals were carried out. However, no Mtb‐FGD2‐sugar or ternary Mtb‐FGD2:F_420_‐sugar complex crystal structures could be obtained. Hence, computational docking was used to provide insights into FGD2:F_420_:D‐glucose ternary complex. Only the β‐anomer of D‐glucose docked in a catalytically relevant mode (binding affinity = −5.275 kcal/mol, ligand efficiency = 0.44), where the glucose C1 atom to the F_420_ C5 atom distance of 3.4 Å is compatible with hydride transfer (Figure 7). Glu51, His78, and Trp82 are also within hydrogen bond distances (3.3, 3.2 and 3.3 Å respectively) of the β‐D‐glucose modeled, suggesting these residues participate in the general acid–base catalysis and hydride transfer from the C1 (anomeric) carbon of the bound sugar to the C5 of the F_420_. The docked substrate pose was used to create a dimeric Mtb‐FGD2:F_420_ ternary complex model for MD simulation. The two active site glutamic acids, Glu147 and Glu263, were predicted to have elevated pK_a_ and were protonated in the MD simulation model. A 200 ns unconstrained MD simulation was run following equilibration. Representative structures for the two active sites were defined as the MD snapshots with the lowest RMSD for active site residues relative to the average structure following structural alignment to the protein non‐hydrogen atoms. In the latter, C2 and C4 hydroxyl groups of β‐D‐glucose make hydrogen bonds with Gln231 and Glu263. These reveal the ligand and cofactor display little conformational sampling with short C‐H and O‐H distances ideal for hydride and proton transfer (Figure 7).

### 2.8 Kinetics of Sugar Oxidation by Mtb‐FGD2

**FIGURE 8:**
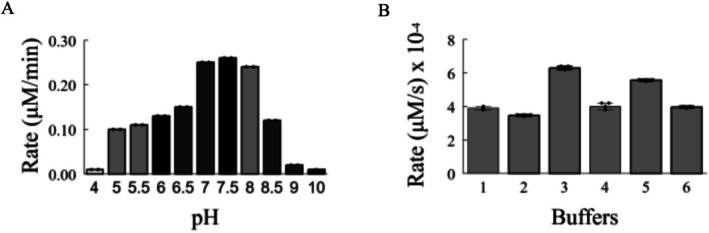
D‐glucose F_420_ reduction pH dependency. (A) Effect of pH on D‐glucose dependent F_420_ reduction, revealing a single optimum pH at 7.5. (B) Effect of 6 buffers (listed in Table 2) on D‐glucose dependent F_420_ reduction. Samples were incubated for 1 h, and the reactions initiated by adding D‐glucose. Reaction rate was determined by monitoring the decrease in absorbance at 420 nm over 5 min at room temperature.

**FIGURE 9:**
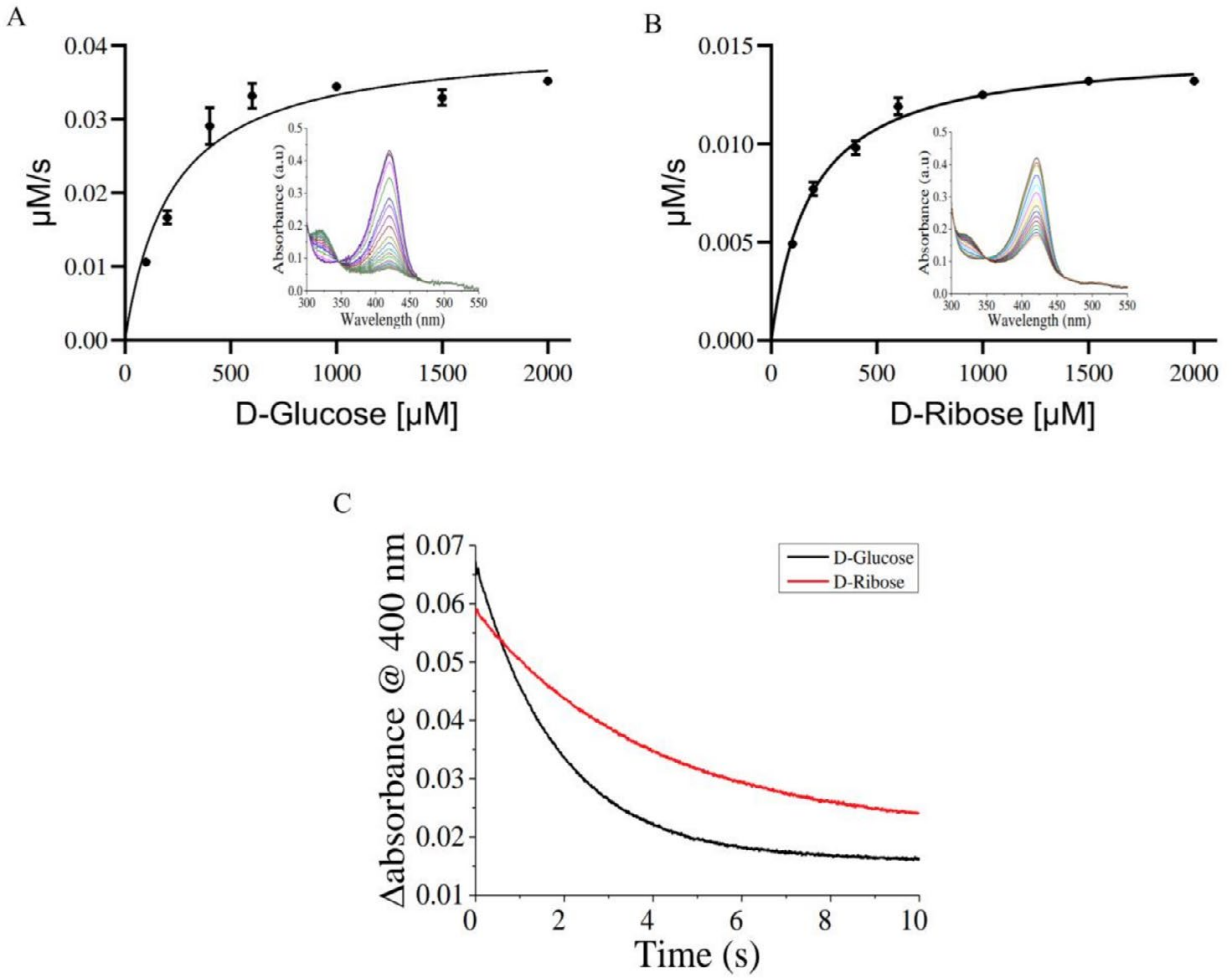
Enzyme kinetics for Mtb‐FGD2. (A) Steady state kinetics and changes in absorbance of Mtb‐FGD2 with D‐glucose and (B) D‐ribose as a function of time (over 1 h). The *K*_m_ of Mtb‐FGD2 for D‐glucose and D‐ribose were estimated to be 220 ± 20 μM and 190 ± 8.0 μM, and *k*_cat_ of 0.40 ± 0.07 s^−1^ and 0.15 ± 0.01 s^−1^ respectively. (C) Pre‐steady state kinetics of Mtb‐FGD2 vs. D‐glucose and D‐ribose, obtained with rapid mixing of 25 μM Mtb‐FGD2, 2.5 μM F_420_ and 2 mM substrate using stopped flow. Data were obtained in quadruplicate and fitted to a single exponential equation to get the following rate constants: *K* = 0.52 ± 0.01 s^−1^ and 0.22 ± 0.01 s^−1^for D‐glucose and D‐ribose respectively.

In the presence of F_420_, Mtb‐FGD2 showed a single optimum pH of 7.5 (Figure 8A) using D‐glucose as the substrate (Buffer No 3, Table 2), with a rate of 6.3 ± 0.1 × 10^−4^ μM/s. Subsequent assays and experiments were carried out using this buffer. Using steady state kinetics, a *K*_m_ value of 220 ± 20 μM, *k*_cat_ of 0.40 ± 0.07 s^−1^, and *k*_cat_/*K*_m_ of 1800 M^−1^ s^−1^ were determined for D‐glucose, and a *K*_m_ value of 190 ± 8.0 μM, *k*_cat_ of 0.15 ± 0.01 s^−1^, and *k*_cat_/*K*_m_ of 800 M^−1^ s^−1^ for D‐ribose (Table 3). The observed rate versus substrate concentration data for both sugars were fitted to the Michaelis–Menten equation using non‐linear regression. Additionally, a pre‐steady state kinetics analysis using stopped flow measurements was carried out for Mtb‐FGD2 in the presence of F_420_ and D‐glucose to determine whether F_420_ exchange or redox chemistry are rate‐limiting. These results were compared with equivalent measurements for D‐ribose. For each substrate, three different mixing regimes were tested: (i) enzyme + cofactor mixed with substrate; (ii) enzyme + substrate mixed with cofactor; (iii) enzyme mixed with cofactor + substrate. For activity against D‐glucose, the results (Figure 9C) showed similar rate constants for the three regimes (*k*_obs_ = 0.54 ± 0.01 s^−1^; 0.49 ± 0.01 s^−1^; 0.52 ± 0.01 s^−1^, respectively), and for D‐ribose (*k*_obs_ = 0.22 ± 0.03 s^−1^, 0.22 ± 0.01 s^−1^, 0.21 ± 0.01 s^−1^ respectively). These results are similar to the *k*_cat_ values obtained using steady state kinetics (Figure 9A,B; Table 3) and indicate that F_420_ exchange at the enzyme active site is unlikely to be rate‐limiting under steady state conditions.

**TABLE 2:** Effects of different buffers on Mtb‐FGD2 activity.

S/N	Buffer	Rate (×10^−4^) μM/s
1	100 mM KPi, 750 mM KCl, 10% glycerol (pH 8.0)	3.9 ± 0.1
2	25 mM KPi (pH 7.0)	3.5 ± 0.1
3	20 mM HEPES, 150 mM NaCl, 1 mM TCEP (pH 7.5)	6.3 ± 0.1
4	50 mM KPi, 20 mM imidazole, 350 mM KCl (pH 8.0)	4.0 ± 0.2
5	50 mM KPi, 150 mM KCl (pH 7.0)	5.6 ± 0.1
6	100 mM KPi, 2 mM NaCl (pH 7.4)	4.0 ± 0.1

*Note:* *k*_obs_ measurements are the mean of 2 repeats.

Abbreviation: KP_i_ = potassium phosphate.

**TABLE 3:** Steady state and pre‐steady state kinetic data for Mtb‐FGD2 against D‐glucose and D‐ribose.

	*K*_m_ (μM)	*k*_cat_ (s^−1^)	*k*_cat_/*K*_m_ (M^−1^ s^−1^)	*k*_1_ (s^−1^) (pre‐steady state)
Mtb‐FGD2 vs. D‐Glucose	220 ± 20	0.40 ± 0.07	1800	0.52 ± 0.01
Mtb‐FGD2 vs. D‐Ribose	190 ± 8.0	0.15 ± 0.01	800	0.22 ± 0.01

### 2.9 Coupling of D‐Glucose‐Dependent F_420_ Reduction by Mtb‐FGD2 to Tfu‐Fno

**FIGURE 10:**
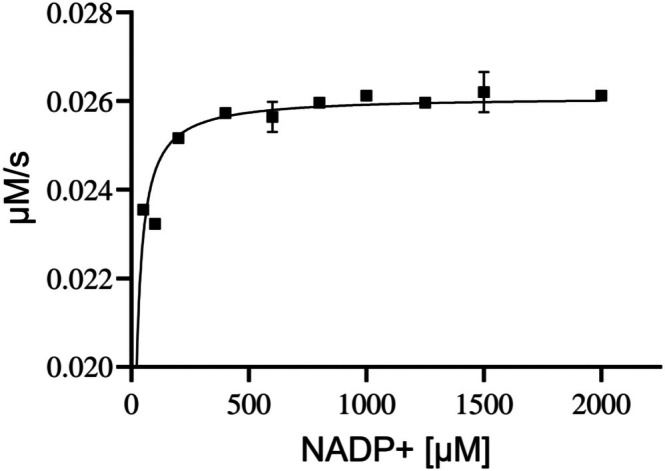
Mtb‐FGD2‐mediated D‐glucose oxidation supports NADP+ reduction by Tfu‐FNO. Plot of the mean observed reaction rates (*k*_obs_) versus the NADP+ concentration of Mtb‐FGD2 and F_420_ mixed with Tfu‐FNO and initiated with 2 mM D‐glucose.

To test whether the reduced F_420_ produced by Mtb‐FGD2 catalyzed D‐glucose oxidation can be utilized by another enzyme, the Mtb‐FGD2 catalytic reaction was coupled to Tfu‐FNO mediated NADP^+^ reduction. Tfu‐FNO is a thermostable F_420_:NADPH oxidoreductase from *T. fusca* [24]. The result shows that Mtb‐FGD2 is able to support an oxidation–reduction reaction in the presence of an F_420_.H_2_‐utilizing enzyme (Figure 10). The reaction consisted of Mtb‐FGD2 (10 μM), F_420_ (10 μM), Tfu‐FNO (200 nM) and varied amounts of NADP+ (50–2000 μM) and was initiated with the addition of 2 mM D‐glucose. By monitoring the increase in absorbance associated with NADPH production at 340 nm, the rate of NADP^+^ consumption was obtained. These rates were plotted against the NADP^+^ concentration and were consistent with a Michaelis–Menten model, resulting in a *K*_m_ value of 7.0 ± 1.2 μM, *k*_cat_ of 0.13 s^−1^, *k*_cat_/*K*_m_ of 18 000 M^−1^ s^−1^ for the D‐glucose‐driven, Mtb‐FGD2‐dependent coupled reaction (Figure 10). The *K*_m_ value obtained is similar to that reported for Tfu‐FNO versus NADPH (7.3 μM), but a significantly lower *k*_cat_ is observed than previously reported (3.3 s^−1^) [24].

## 3 Discussion

The results demonstrate high‐level heterologous expression of Mtb‐FGD2 is feasible when using GroEL/ES‐expressing in *E. coli*, supporting purification of active Mtb F_420_‐dependent dehydrogenase Mtb‐FGD2 and the determination of crystal structures in *apo* and F_420_‐complex forms. Overall, the protein assumes the classical TIM‐barrel architecture and shows the characteristic *cis*‐peptide bond seen in other F_420_‐binding enzymes such as Mtb‐FGD1 [10], Mer [17], Adf [20], and Rh‐FGD [18]. Similar to other enzymes with a TIM‐barrel structure, the Mtb‐FGD2 active site lies within the C‐terminal end of the eight parallel β‐strands [25]. The 3‐membered F_420_ aromatic ring system is buried deep in the active site, while the tail extends towards the solvent. The considerably tighter binding of the cofactor in Mtb‐FGD2 cannot be directly accounted for by comparison of the Mtb‐FGD1 and Mtb‐FGD2 F_420_ complex structures, which are remarkably similar in the extent and nature of the protein‐ligand interactions. However, we speculate that the increased Mtb‐FGD2 affinity might be an adaptation to the distinct conditions outside the inner membrane.

Mtb‐FGD2 is shown to become significantly more thermostable in the presence of F_420_. The relatively low melting temperature of the *apo*‐form may partly explain why it is hard to obtain its crystal structure without the use of the additive, TEW, which possesses the ability to enhance protein crystal packing and stability without altering functionality [14, 16]. This approach of utilizing an additive such as TEW for the crystallization of gene products has been previously documented, including for the crystallization of the nuclear binding domain of HSP70 [16], lysozyme [14], and mushroom tyrosinase [15]. Two of the residues of Mtb‐FGD2 predicted to play a role in the binding of the hydroxybenzyl moiety of F_420_, thereby positioning it for substrate oxidation, are Ala232 and Glu263 [19]. Examination of the F_420_‐bound Mtb‐FGD2 indicates that whereas Glu263 lies within hydrogen bond distance of a water molecule that interacts with the bound F_420_, it is Gln231 that makes a favorable hydrogen bond interaction with the cofactor, as opposed to Ala232.

**FIGURE 11:**
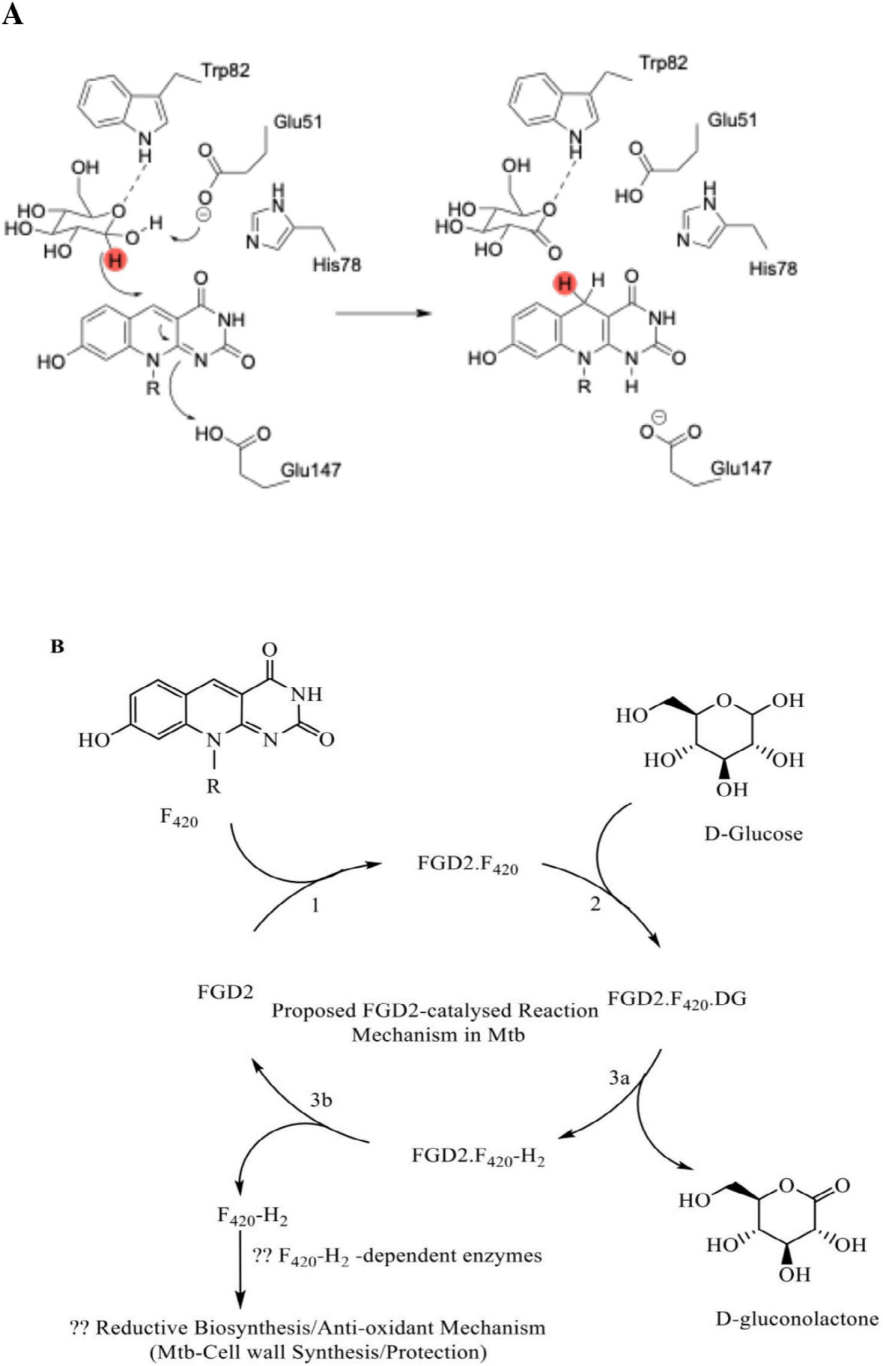
Proposed reaction mechanism for Mtb‐FGD2 oxidation of D‐glucose. (A) Active site general acid–base catalysis and hydride transfer involving Glu51, His78, Trp82 and Glu147 and conversion of F_420_ to F_420_.H_2_. (B) Proposed reaction pathway for the oxidation of D‐glucose to D‐gluconolactone and reduction of F_420_ to F_420_.H_2_. Mtb cell wall enzymes for the utilization of F_420_.H_2_ are yet to be identified, but possible candidates include F_420_.H_2_‐dependent phthiodiolone ketoreductase [19] and quinolone reductase [28], both of which have been shown to be cell envelope‐bound, and present only in members of pathogenic *Mycobacterium tuberculosis* complex, such as Mtb, where they play a role either in the cell wall biosynthesis or in the anti‐oxidant mechanisms that protect the bacteria against oxidative stress.

While Mtb‐FGD2 lacks activity with G6P, the crystal structure reveals a predominantly polar active site that appears incompatible with the long hydrocarbon tail of proposed HMA substrate. Additionally, enzymes that accommodate longer aliphatic R‐tail from hydroxy mycolic acid‐like substrates, such as the *Mycobacterium tuberculosis* β‐ketoacyl Acyl Carrier Protein (ACP) Synthase I (Mtb‐KasA) [26] and β‐Ketoacyl ACP Synthase II (Mtb‐KasB) [27], tend to have larger substrate cavities unlike Mtb‐FGD2. Given the conservation of many active site elements with Mtb‐FGD1, but notably not those implicated in the phosphate binding site, we postulated a simple sugar could act as substrate. Indeed, D‐glucose and structurally related sugars were shown to support F_420_ reduction. While efforts at determining the crystal structure of a ternary complex with F_420_ and a sugar substrate were unsuccessful, computational docking and molecular dynamics suggested β‐D‐glucose can bind in a catalytically relevant conformation. The similarity of the Mtb‐FGD2 active site with Mtb‐FGD1 suggests a similar catalytic mechanism as Mtb‐FGD1 [10] and Adf [20], consisting of general acid–base catalysis, hydride and proton transfer involving His78, Trp82, Glu51, and Glu147 (Figure 11A).

The localization of FGD2 to the mycobacterial cell wall [11] suggests a putative role as a glucose‐dependent source of reduced F_420_ for F_420_.H_2_ dependent enzymes that are involved in cell wall synthesis and, potentially, a defense mechanism against host‐generated oxidative stress (Figure 11B). Recent studies have demonstrated early‐secreted antigenic factor (ESAT‐6) mediated overproduction of glucose in Mtb‐infected human macrophages [29]. ESAT‐6 is a virulent factor that is present only in pathogenic *Mycobacterium tuberculosis* complex (MTBC) such as Mtb, *M. bovis* , *M. leprae* , and *M. kansasa*, and has been widely used as an important biomarker for Mtb diagnosis [30]. By binding to GLUT‐1 in the host, it has been shown to induce perturbations in the metabolic flux that ultimately result in the formation of foamy macrophages, thus providing a safe haven for mycobacterial survival [29]. This points to the host glucose serving as the physiological substrate for FGD2‐mediated reduction of F_420_ for Mtb in this cellular compartment, generating reduced F_420_ for other cell surface‐bound F_420_.H_2_‐dependent enzymes. Interestingly, a few examples of such enzymes have been reported, such as phthiodiolone ketoreductase (PKR) [19], and Rv1261c [28], an F_420_.H_2_‐dependent quinone reductase that has been localized to the Mtb cell envelope. Similar to the genes encoding ESAT‐6 and Mtb‐FGD2 (Rv0131c), PKR‐encoded genes are conserved only in pathogenic MTBC species, while absent in non‐pathogenic ones like *M. smegmatis* , and it is involved in the synthesis of apolar lipids called phthiocerol dimycocerosates (PDIM) [19]. PDIM is a glycolipid virulence factor that has been demonstrated, in concert with ESAT‐6, to facilitate Mtb entry into the cytosol of human lymphatic endothelial cells via disruption in their phagosomal membranes [31].

We hypothesize that cell‐wall localized Mtb‐FGD2 utilizes host D‐glucose to provide reducing equivalents in the form of F_420_.H_2_. In vitro D‐glucose driven Mtb‐FGD2 coupled reactions with NADP^+^/F_420_‐dependent Tfu‐FNO support this hypothesis. In vivo, the Mtb‐FGD2 reduced F_420_ produced can be utilized for the two‐electron reduction of exogenous quinones to dihydroquinones, a putative anti‐oxidant mechanism that serves to protect against oxidative stress [28], the metabolism of endogenous membrane‐bound menaquinone [28] or for Mtb cell wall synthesis through the formation of PDIM [31].

While the fate of D‐gluconate produced by the spontaneous hydrolysis of D‐gluconolactone product is unclear, we postulate that an alternative oxidative phase of the pentose phosphate pathway (AOXPPP), similar to that identified in *Cryptococcus neoformans* [32], might be present in Mtb. Indeed, the gluconate shunt is present mostly in plants and algae, but they have been identified also in cyanobacteria, and a number of Gram‐negative and Gram‐positive bacteria that utilize the Entner–Doudoroff pathway for the degradation of glucose [33]. In the gluconate shunt, D‐gluconate is either oxidized by gluconate dehydrogenase to 2‐keto‐3‐deoxygluconate followed by further degradation to D‐glyceraldehyde and pyruvate by 2‐keto‐3‐deoxygluconate aldolase, or catabolized by gluconate kinase to 6‐phosphogluconate, an important intermediate in the Entner–Doudoroff pathway, before being ultimately converted to the same end products as above, namely D‐glyceraldehyde and pyruvate, by the activity of 2‐keto‐3‐deoxy‐6‐phosphogluconate aldolase [33]. This pathway allows for the utilization of gluconate as an alternative carbon source via its conversion to 6‐phosphogluconate in the presence of gluconate kinase [32, 33]. Interestingly, an Entner–Doudoroff pathway of gluconate utilization has also been identified in a species of antibiotic‐producing actinomycete [34]. Depending on the prevailing circumstance such as oxygen tension and nutrient level, the released pyruvate could be converted to lactate via lactate dehydrogenase, or channeled into the tricarboxylic acid cycle for ATP generation via oxidative phosphorylation or converted to alanine for further amino acid synthesis or simply utilized as a precursor for new glucose synthesis through gluconeogenesis, among other possibilities [35].

## 4 Experimental Procedures

### 4.1 Cloning, Expression and Purification of Mtb‐FGD2 (Rv0132c)

The gene encoding the Mtb‐FGD2 open reading frame, Rv0132c, was synthesized as an N‐terminal TEV‐cleavable 6×‐histidine construct, without the 1st 30 amino acids at the N‐terminal, being the residues representing the twin‐arginine translocation (Tat) signal sequence [11, 36]. The construct was PCR‐amplified and subsequently cloned into pET24b by in‐fusion technology (Takara Bio). The primers were designed to have 15‐bp extensions that are complementary to the ends of the linearized plasmid (Table 4).

**TABLE 4:** Summary of primers used for the expression of Mtb‐FGD2 in *E. coli* .

*Gene*	*Forward Primer* (*5*′ *– 3*′)	*Reverse Primer* (*5*′ *– 3*′)
Rv032c (Mtb‐FGD2)	*TAT TTT CAG GGT AGC* AGC GGT CCG ACA CCG ACA	*AGC AGC CGG ATC TCA* TCA ACG CAG TTC CGG CAG AAC AT
pET24b Vector	*GCT ACC CTG AAA* ATA CAG ATT TTC AC	*TGA GAT CCG GCT GCT* AAC AAA G

*Note:* Italicised oligonucleotide sequences represent the 15‐bp complementary extensions.

The presence of the gene of interest was confirmed with colony PCR and gene sequencing. This was followed by transformation into competent *E. coli* cells BL21 (DE3) containing a cold‐adapted T7‐polymerase driven IPTG‐inducible GroEL/ES plasmid (graciously supplied by Dr. Stephen Marshall, Manchester Institute of Biotechnology). Cells were grown in 2xYT media at 37°C with 30 μg/mL kanamycin and 50 μg/mL streptomycin for 1 h, then at 30°C until a log phase of OD_600_ of 0.6–0.8 was reached, at which point the temperature was reduced to 16°C. Induction of protein expression was achieved with 400 μM IPTG (isopropyl β‐d‐1‐thiogalactopyranoside) at 16°C while cell culture was continued at the same temperature overnight on a shaker. Cells were harvested by centrifugation (5000 × g, 4°C × 15 min), and lysed using a Bandelian Sonicator (for 30 min with each cycle set as 15 s on– 45 s rest, 38% amplitude, 4°C) in buffer containing 50 mM HEPES, 150 mM NaCl, at pH 7.5. Purification was achieved first by using gravity columns with IMAC, namely Ni‐nitrilotriacetic acid (Ni‐NTA) [37] After desalting and an overnight cleavage with tobacco etch virus (TEV) protease, the protein was reversed‐purified with the same IMAC and the sample obtained was then concentrated to a final volume of ~1 mL (with Vivaspin 30 concentrator, Generon) [38] prior to further purification on AKTA Pure using HiLoad 16/600 Superdex 200 pg (GE Healthcare). The column was pre‐equilibrated with SEC buffer (pH 7.5) and protein purification achieved using the gel filtration method and stored in the same buffer at −80°C until further use. Protein purity was achieved with SDS PAGE, while its concentration was estimated by measuring its absorbance at 280 nm wavelength on a Nanodrop instrument (NanoDrop Technologies) using the Beer– Lambert equation, namely *A = εbc* (A is the absorbance, *ε*, the extinction coefficient of the protein, *b* is the path length and *c*, the concentration of the protein), as previously described [37]. The extinction coefficient was obtained by inputting the protein sequence into the protein parameter estimation tool, ExPASy‐ProtParam. Crystals for both the ligand‐free and F_420_‐bound Mtb‐FGD2 were obtained using the sitting‐drop vaporization diffusion method [16, 18, 39, 40]. Approximately 300 nL of freshly prepared protein sample in SEC buffer containing 22 mg/mL Mtb‐FGD2, 5 mM TEW with or without approximately 1 mM F_420_, was delivered in drops into an equal amount of MORPHEUS crystallization screen using the nanoliter protein crystallization robot (Mosquito Xtal3, SPT Labtech) at 20°C and incubated at 4°C.

### 4.2 Native MS Sample Preparation and Acquisition

Approximately 10 μL of Mtb‐FGD2 (10 μM final concentration) was buffer‐exchanged into 150 mM ammonium acetate (pH 7.0) using Zeba Micro Spin Desalting Columns 7 kDa MWCO according to manufacturers' instructions (Thermo Scientific, catalogue number 89878). Data were acquired on a Thermo UHMR mass spectrometer operating in positive mode using the vendor software Tuneplus 2.10, build 2990 (Thermo Fisher Scientific, Hemel Hempstead, UK). Nano‐ESI capillaries were prepared in‐house from thin‐walled borosilicate capillaries (inner diameter 0.9 mm, outer diameter 1.2 mm, World Precision Instruments, Stevenage, UK) using a Laser micropipette puller (Sutter Instrument Company, Novato, CA, USA). A positive voltage was applied to the solution via a platinum wire (Goodfellow Cambridge Ltd., Huntington, UK) inserted into the capillary. Gentle conditions were used to preserve the native‐like structure: spray voltage 1.2–1.5 kV and Nitrogen supplied in‐house was used as the carrier gas. External mass calibration of the spectra was achieved using solutions of cesium iodide (2 mg/mL in 50:50 water: isopropanol). Data were processed using Thermo Scientific Xcalibur 4.1.31.9, Qualbrowser (Thermo Fisher Scientific, Hemel Hempstead, UK). Deconvolution of acquired data was carried out using the application UniDec 4.2.0 [41] and data were also exported from Qualbrowser into the Opensource application Inkscape 1.3 for annotation.

### 4.3 Data Collection and Structure Determination of Apo‐ and F_420_ ‐Bound Mtb‐FGD2

The screen condition contained premixed crystallization additives, cryo‐protectant and buffer systems [21] so no additional cryo‐protectant was required before the crystals were put in liquid nitrogen and shipped to Beamline i03, Oxford (UK) for X‐ray diffraction data collection. Data sets were collected (synchrotron Block Allocation Group (BAG) number MX31850) at a wavelength of 0.9763 Å, images were scaled and integrated using DIALS (Diffraction Integration for Advanced Light Sources) [42] and data reduction achieved with AIMLESS on the CCP4i2 suite [43]. Mtb‐FGD2 structure was solved by Single‐wavelength Anomalous Diffraction (SAD) technique CRANK2 [44] and refinement, using the reciprocal‐space refinement tool, REFMAC, was carried out in the CCP4i2 suite [43, 45]. The initial model building and refinement, followed by the iterative cycle of rebuilding and refinement, were done in COOT as well as in REFMAC [45]. Electron densities for F_420_ were observed in 2 of the monomers, with an additional un‐modeled blob which was modeled as MPD derived from the screen condition in the active site close to the isoalloxazine ring of F_420_ in each instance. In both the free and complex forms, the final models comprise amino acids ranging from 37 to 360, with *R* = 0.12 and *R*_free_ = 0.15 for *apo*‐FGD2, and *R* = 0.21, *R*_free_ = 0.25 and 2 molecules of F_420_ for the complex form. Details of data processing statistics are as shown in Table 5.

**TABLE 5:** Data collection and refinement statistics.

	Ligand‐free Mtb‐FGD2 (Accession No 9FP4)	F_420_‐bound Mtb‐FGD2 (Accession No 9FFP)
Wavelength (Å)	0.9795	0.9763
Space group	P 2_1_2_1_2_1_	P 2_1_2_1_2_1_
Cell constants	89.86 Å 92.16 Å 100.28 Å	87.31 Å 89.31 Å 155.63 Å
a, b, c, *α*, *β*, *γ*	90.00° 90.00° 90.00°	90.00° 90.00° 90.00°
Resolution (Å)^a^	1.47–1.45 (54.15–1.45)	2.42–2.35 (77.82–2.35)
Completeness (%)^a^	97.9 (80.4)	100 (100)
Overall observations	1 840 968	675 719
Number of reflections (Unique)	144 578	51 484
Overall *R*_meas (all *I*+ & *I*−)_ ^a^	0.172 (1.427)	0.293 (1.088)
Overall *R*_meas (within *I*+ & *I*−)_ ^a^	0.139 (1.427)	0.288 (1.086)
*I*/*σ* ^a^	11.4 (1.4)	6.2 (2.1)
CC Half	0.998	0.996
Multiplicity^a^	12.7 (8.1)	13.1 (11.5)
*R*_fwork_ ^a^	0.12 (0.25)	0.21 (0.29)
*R*_free_ ^a^	0.15 (0.25)	0.25 (0.31)
Rmsd bond length (Å)	0.0135	0.0076
Rmsd bond angle (°)	1.912	1.889
Residues in favored regions (%)	97.00	97.00

^a^ Values in parentheses report on outer bin values.

### 4.4 F_420_ Production and Purification

F_420_ was produced from wild type *M. smegmatis* MC^2^155 cells, grown to high density in a 2 L flask of 2xYT media (containing 0.08% tween 80, 50 μg/mL of ampicillin at 37°C for 72 h in the presence or absence of 0.1% glycerol), using a slightly modified previously documented protocol [46, 47]. Cells were harvested by centrifugation and lysed in 75% ethanol in 25 mM KPi buffer (pH 7.0). Following removal of the ethanol content using a rotatory evaporator, the F_420_‐containing paste was re‐suspended in 25 mM KPi buffer (pH 7.0), applied first unto a pre‐equilibrated Q sepharose column for anion exchange chromatography on AKTA Pure. F_420_ was eluted with 100% buffer B containing 1 M NaCl in 25 mM KPi buffer and further purified with reverse phase HPLC using a C18 column. For the preparatory HPLC, solution A was water only (or with 0.1% formic acid) and solution B was methanol only (or acetonitrile with the same percent of formic acid). The column was first purged for 5 min at 50% solution A and 50% solution B respectively prior to sample application. Fractions from preparatory HPLC were then lyophilized and stored in powdered form. F_420_ concentration was estimated using the extinction coefficient at 420 nm (41.4 mM^−1^ cm^−1^, pH 7.5 at 25°C) as previously documented [47, 48].

### 4.5 F_420_ ‐Binding Affinity Determination

The binding affinity of Mtb‐FGD2 to F_420_ was determined by using Isothermal Titration Calorimetry (ITC) and Tryptophan Fluorescence Quenching Spectroscopy (TFS). Data with ITC were obtained by titrating approximately 60 μL of Mtb‐FGD2 240 μM final concentration, suspended in SEC buffer (pH 7.5) in the syringe against 300 μL of F_420_ (30 μM final concentration, in the same buffer solution as for the enzyme) in the sample cell at 25°C. Data were obtained using the following experimental parameters: 4 s filter period, 19 injections of 2 μL each (0.4 μL for first injection), at an interval of 150 s in between injections, and a reference power of 10 μcal/s. Data were exported to generate ITC thermogram and *K*_d_ estimation, using MicroCal PEAQ‐ITC software on the instrument. For the TFS, spectra were obtained with a 1‐cm path‐length cuvette on a CARY (Varian, UK) Eclipse Fluorescence Spectrophotometer at a constant temperature of 25°C, maintained by a Peltier connected externally as previously documented [49]. The excitation wavelength was set at 285 nm, and emission spectra collected over a range of 300–400 nm wavelength, and a slit width of 5 nm. The reaction mixture of approximately 500 μL contained 1 μM of Mtb‐FGD2 in SEC buffer at a pH of 7.5 and was titrated against with increasing concentrations of F_420_ (0–5 μM) until the intrinsic tryptophan fluorescence was completely quenched, as reported previously [49, 50]. Data were exported as an excel file into OriginPro (version 9.1 64Bit). The observed change in fluorescence associated with each F_420_ concentration, relative to that of Mtb‐FGD2 only, was plotted against F_420_ concentration and the *K*_d_ was estimated by fitting the curve to the tight binding non‐linear model in OriginPro.

### 4.6 UV–Vis Spectroscopy Assays and Substrate Profiling

The activity of Mtb‐FGD2 was tested against a number of simple sugars (such as D‐glucose, D‐ribose, D‐galactose, etc.), derived sugars (such as G6P, D‐Glucosamine 6‐Phosphate, Ribose‐5 phosphate, etc.) and some hydroxylated compounds (such as N‐acetylglucosamine, trehalose, D‐lactate, and isopropanolol). All assays were carried out in 1‐cm path length cuvette on CARY 60 UV–vis spectrophotometer (Varian, UK). The reaction mixture (200 μL) contained 100 nM Mtb‐FGD2, 5–10 μM F_420_ and 1–2 mM substrate in SEC buffer. Each reaction was started by the addition of substrate, and spectra obtained (over 200–800 nm wavelength) at 0 and 1 min, then at an interval of 5 min for 1 h. Where significant activities were observed, steady state kinetic parameters were obtained at 25°C by varying substrate concentrations while keeping enzyme and F_420_ concentrations constant using the same buffer as for activity assay. Typically, approximately 100 nM Mtb‐FGD2, 10 μM F_420_ and 0–2 mM substrate were suspended in the SEC buffer (pH 7.5) at a total volume of 200 μL in each case. Data were exported in Excel format and analyzed on OriginPro to obtain *K*_m_, V_max_ using Michaelis–Menten equation and from which *k*_cat_ and *k*_cat_/*K*_m_ ratio were calculated.

### 4.7 Optimum pH and Buffer Determination

Optimum pH for Mtb‐FGD2 activity was determined using the sugar for which the enzyme has the greatest catalytic efficiency, namely D‐glucose. Reaction mixture contained 100 nM enzyme and 10 μM F_420_ in 20 mM HEPES, 150 mM NaCl, 1 mM TCEP, at a pH range of 4–10. After incubation for 1 h at room temperature, each reaction was initiated with 2 mM D‐glucose and monitored by following the decrease in absorbance of F_420_ at its isosbestic point (401 nm) over 10 min on a CARY 60 UV–Vis spectrophotometer (Varian, UK) at 25°C. The reaction rate (change in absorbance over time) was used as a function of Mtb‐FGD2 activity at each pH. The enzyme activity was similarly tested in six buffer systems (containing different forms of additives such as glycerol, imidazole, NaCl, KCl, and TCEP, and at a pH range of 7–8). Approximately 1 μM of the enzyme was mixed with 10 μM of F_420_ and reaction initiated with 3.2 mM D‐glucose. By monitoring the decrease in absorbance at 420 nm, the amount of substrate consumed per unit time was calculated using the extinction coefficient of F_420_ at 25°C (41.4 mM^−1^ cm^−1^).

### 4.8 Mtb‐FGD2 Thermal Stability

The thermo‐stability of free Mtb‐FGD2 and in the presence of F_420_ was tested using the static light scattering (SLS) method on the protein stability screening platform UNcle (Unchained Labs). All samples were suspended in SEC buffer at a pH of 7.5. For the ligand‐free protein, approximately 14 μM of the enzyme was suspended in SEC buffer, while a five‐fold concentration (70 μM) of the ligand (F_420_) was added to the protein for the ligand‐containing mixture. Data (*T*_m_1 values, Tagg, etc.) were obtained in duplicates at a temperature of 25°C by loading approximately 8.5 μL of the sample mixture into the 16‐well UNcle cassette.

### 4.9 Molecular Docking

Molecular docking was performed using AutoDock Tools with a default setting [51, 52] following the method outlined by Forli et al. [53]. The coordinates of the *α*‐ and *β*‐ anomers of the D‐glucose and D‐ribose sugars (constituting the ligand) were obtained by running their respective canonical smiles on CCP4i2 software (version 7.1.010) [42] while those of the F_420_‐bound protein (the receptor) were obtained from our F_420_‐bound FGD2 crystal structure. Ligand preparation was performed using AutoDock Vina (version 1.5.7) and saved as a PDBQT file after ligand root detection and choice of torsion [53]. The receptor was prepared first by removing all the water molecules and the crystallization additive (TEW). Hydrogens were added using the “noBondOrder” and “renumber atoms to include new hydrogens” options in AutoDock Tools [52]. Gasteiger charges were added automatically. The search grid box was set such that it covered the entire protein‐ligand complex observed in our F_420_‐bound FGD2 crystal structure. To ensure better sampling of the possible binding sites, the exhaustiveness was set at 24. Each of the best 9 binding poses was analyzed separately and visualized in Pymol for assessment of their binding modes and chemistry.

### 4.10 Molecular Dynamics Simulations

The docked substrate pose shown in Figure 7 was used to create a model for the MD simulation. The cofactor and ligand were added to the second monomer of the enzyme by copying the coordinates for the cofactor and ligand after structural alignment of the two monomers. Propka 2.0 [54] was used to calculate the pK_a_ values of titratable residues. The model was solvated in a TIP3P water box with sides 10 Å longer than the enzyme, with 18 Na^+^ counter ions to balance the charge, for a total of 29 088 atoms. Generalized Atom Force field (GAFF) parameters for F_420_ and glucose were generated using the AM1‐BCC method in Antechamber [55], and the Amber ff14SB force field [56] was used for the water molecules and Na^+^ counter ion. MD simulations were performed using Gromacs 2020.3 [57, 58] with LINCS bond constraints applied to all bonds involving hydrogen [59] Verlet integration, 10 Å cut‐offs for Coulombic and van der Waals interactions [60] Particle Mesh Ewald for long‐range electrostatics, the velocity‐rescaling modified Berendsen thermostat (300 K), the Parrinello‐Rahman barostat [61] (1 bar), periodic boundary and a 2 fs timestep. Simulations were run according to the following protocol: (i) energy minimisation (ii) 1 ns constant volume (NVT) equilibration with position restraints with force constants of 10 kJ mol^−1^ Å^−1^ on the non‐hydrogen atoms of the enzyme; (iii) 1 ns constant pressure (NPT) equilibration of the solvent with the same position restraints; (iv) 1 ns constant pressure (NPT) equilibration with position restraints with force constants of 1 kJ mol^−1^ Å^−1^ (v) 200 ns unconstrained MD simulation. Representative structures for the two active sites were defined as the MD snapshots with the lowest RMSD for active site residues relative to the average structure following structural alignment to the protein non‐hydrogen atoms (see Figure 7).

## Author Contributions

**Adewale V. Aderemi:** investigation, writing. **Matthew Snee:** investigation, writing – review and editing, validation. **Richard B. Tunnicliffe:** investigation. **Linus O. Johanissen:** investigation, methodology, writing – review and editing. **Matthew J. Cliff:** validation, methodology. **Colin W. Levy:** validation. **Derren J. Heyes:** validation, methodology. **Marina Golovanova:** investigation. **Thomas A. Jowitt:** investigation, validation. **Sam Hay:** investigation, validation. **Andrew W. Munro:** funding acquisition, project administration, supervision. **Jonathan P. Waltho:** supervision, writing – review and editing, validation, funding acquisition. **David Leys:** project administration, writing – review and editing, supervision.

## Funding

AVA received funding from Tertiary Education Trust Fund (TETFund), Nigeria. The work was supported by BBSRC grants BB/X019217/1 to JPW and BB/R009961/1 and to BB/I019227/1 AWM. We thank Diamond Light Source for access (proposal number: MX12788 and MX31850) for the data collection.

## Conflicts of Interest

The authors declare no conflicts of interest.

## Supporting information


**Table S1:** Generalized amber force field (GAFF) parameters for D‐glucose.
**Table S2:** Generalized amber force field (GAFF) parameters for F420.
**Figure S1:** Atom numbering for D‐Glucose GAFF parameters in Table S1.
**Figure S2:** Atom numbering for F420 GAFF parameters in Table S2.

## Data Availability

Structural data and associated structure factors can be accessed via the Protein Data Bank using the Accession Numbers 9FP4 and 9FFP.
