# Supplementary material for: The Mycobacterium tuberculosis Rv0132c Gene Product Mtb‐FGD2 Can Act as an F420 ‐Dependent Glucose Dehydrogenase
Source: Proteins. 2026 Apr 21;94(9):1610–27. doi: 10.1002/prot.70139 (PMC13434993; doi:10.1002/prot.70139)
Supplement: Supplementary file 1 [file PROT-94-1610-s001.docx]

**Table S1** Generalized Amber Force Field (GAFF) parameters for D-glucose.

| Atom #^1^ | Name | Atom type | Charge |
| --- | --- | --- | --- |
| 1 | O1 | oh | -0.5938 |
| 2 | H1 | ho | 0.414 |
| 3 | C1 | c3 | 0.1394 |
| 4 | H7 | h1 | 0.0642 |
| 5 | H9 | h1 | 0.0642 |
| 6 | C2 | c3 | 0.0781 |
| 7 | H | h1 | 0.0587 |
| 8 | O2 | os | -0.4606 |
| 9 | C3 | c3 | 0.3479 |
| 10 | O3 | oh | -0.5628 |
| 11 | H6 | ho | 0.416 |
| 12 | H2 | h2 | 0.0977 |
| 13 | C4 | c3 | 0.0621 |
| 14 | O4 | oh | -0.5788 |
| 15 | H8 | ho | 0.421 |
| 16 | H3 | h1 | 0.0467 |
| 17 | C5 | c3 | 0.0661 |
| 18 | O5 | oh | -0.5868 |
| 19 | H10 | ho | 0.411 |
| 20 | H4 | h1 | 0.0897 |
| 21 | C6 | c3 | 0.0841 |
| 22 | H5 | h1 | 0.0897 |
| 23 | O6 | oh | -0.5768 |
| 24 | H12 | ho | 0.409 |

^1^See Figure S1 for atom numbering

Table S2 Generalized Amber Force Field (GAFF) parameters for F420.

| Atom #^1^ | Name | Atom type | Charge | Atom # | Name | Atom type | Charge |
| --- | --- | --- | --- | --- | --- | --- | --- |
| 1 | C12 | ca | -0.047 | 44 | O2 | o | -0.8335 |
| 2 | H4 | ha | 0.129 | 45 | O3 | o | -0.8335 |
| 3 | C11 | ca | -0.192 | 46 | O1 | os | -0.5792 |
| 4 | H3 | ha | 0.155 | 47 | C2 | c3 | 0.1301 |
| 5 | C10 | ca | 0.2261 | 48 | C1 | c3 | -0.1181 |
| 6 | O8 | oh | -0.4871 | 49 | H | hc | 0.041033 |
| 7 | H31 | ho | 0.482 | 50 | H1 | hc | 0.041033 |
| 8 | C9 | ca | -0.284 | 51 | H2 | hc | 0.041033 |
| 9 | H24 | ha | 0.203 | 52 | H6 | h1 | 0.0587 |
| 10 | C8 | ca | 0.1407 | 53 | C19 | c | 0.6421 |
| 11 | C13 | ca | -0.199 | 54 | O11 | o | -0.6711 |
| 12 | C14 | cc | 0.068 | 55 | N4 | n | -0.5049 |
| 13 | H5 | ha | 0.156 | 56 | H26 | hn | 0.3295 |
| 14 | C15 | cd | -0.2896 | 57 | C20 | c3 | -0.0323 |
| 15 | C16 | c | 0.7261 | 58 | C29 | c | 0.9106 |
| 16 | O9 | o | -0.6725 | 59 | O17 | o | -0.8508 |
| 17 | N2 | n | -0.6155 | 60 | O18 | o | -0.8508 |
| 18 | H25 | hn | 0.3275 | 61 | H7 | h1 | 0.0657 |
| 19 | C17 | c | 0.8529 | 62 | C21 | c3 | -0.0674 |
| 20 | O10 | o | -0.6325 | 63 | H8 | hc | 0.0557 |
| 21 | N3 | nc | -0.4364 | 64 | H9 | hc | 0.0557 |
| 22 | C18 | cd | 0.2157 | 65 | C22 | c3 | -0.1474 |
| 23 | N1 | na | -0.0831 | 66 | H10 | hc | 0.0517 |
| 24 | C7 | c3 | -0.0117 | 67 | H11 | hc | 0.0517 |
| 25 | H22 | h1 | 0.0962 | 68 | C23 | c | 0.6421 |
| 26 | H23 | h1 | 0.0962 | 69 | O12 | o | -0.6731 |
| 27 | C6 | c3 | 0.1281 | 70 | N5 | n | -0.5199 |
| 28 | O7 | oh | -0.6068 | 71 | H27 | hn | 0.3065 |
| 29 | H30 | ho | 0.418 | 72 | C24 | c3 | -0.0333 |
| 30 | H21 | h1 | 0.0917 | 73 | C28 | c | 0.9056 |
| 31 | C5 | c3 | 0.0981 | 74 | O15 | o | -0.8633 |
| 32 | O6 | oh | -0.6438 | 75 | O16 | o | -0.8633 |
| 33 | H29 | ho | 0.453 | 76 | H12 | h1 | 0.0647 |
| 34 | H20 | h1 | 0.0867 | 77 | C25 | c3 | -0.0474 |
| 35 | C4 | c3 | 0.1001 | 78 | H13 | hc | 0.0337 |
| 36 | O5 | oh | -0.6948 | 79 | H14 | hc | 0.0337 |
| 37 | H28 | ho | 0.495 | 80 | C26 | c3 | -0.1744 |
| 38 | H19 | h1 | 0.0307 | 81 | H15 | hc | 0.0352 |
| 39 | C3 | c3 | 0.1644 | 82 | H16 | hc | 0.0352 |
| 40 | H17 | h1 | 0.0457 | 83 | C27 | c | 0.9026 |
| 41 | H18 | h1 | 0.0457 | 84 | O14 | o | -0.8918 |
| 42 | O4 | os | -0.5752 | 85 | O13 | o | -0.8918 |
| 43 | P1 | p5 | 1.4528 |  |  |  |  |

^1^See Figure S2 for atom numbering

Figure S1. Atom numbering for D-Glucose GAFF parameters in Table S1

Figure S2. Atom numbering for F420 GAFF parameters in Table S2
